# Supplementary material for: An investigation of the impact of human capital and supply chain competitive drivers on firm performance in a developing country
Source: PLoS One. 2022 Dec 1;17(12):e0274592. doi: 10.1371/journal.pone.0274592 (PMC9714865; doi:10.1371/journal.pone.0274592)
Supplement: Attachment — Submitted filename: plosone review report 31052022.docx [file pone.0274592.s001.docx]

Thank you for the opportunity offered me to review the manuscript on “An investigation of the impact of human capital and supply chain competitive drivers on firm performance in a developing country” This summary is the observations made as I reviewed the paper.

**Review Comments**

**Theoretically, the paper was introduced with conceptual framework theorizing the human capital and supply chain drivers in relation with firm’s performance, evidence from specific references from key literatures to support their claims.**

**However reference must be made of recent literatures on the topic which can easily be found and available on google scholars and other search engines.**

**Methodology deployed is online survey used to capture 600 firms as respondents. The SEM was used in the study was was applicable and workable enough. SPSS and AMOS software were used to analyse the data and for the various tests were performed which yielded feasible results.**

**The English Language though was fairly good need to be improved. Figure 2 was referenced in the results and discussions write up but it cannot be found in the paper. I suggest Figure 3 should rather be labeled as figure 2.**

**Fitness; The topic is contemprorary enough and fits into the scope of PLOSONE**

**Therefore, the manuscript need revisions on the issues raised to improve its current form for suitability for publication.**

**References**

**Besides adding additional modern references, the current references must be formatted in line with PLOSONE requirements.**
