# Supplementary material for: An investigation of the impact of human capital and supply chain competitive drivers on firm performance in a developing country
Source: PLoS One. 2022 Dec 1;17(12):e0274592. doi: 10.1371/journal.pone.0274592 (PMC9714865; doi:10.1371/journal.pone.0274592)
Supplement: Attachment — Submitted filename: Answer_reviever.docx [file pone.0274592.s002.docx]

| Issue addressed by reviewers | Answer |
| --- | --- |
| Page2: 2nd paragraph: "98% of country's business" --> What is the metric? | This was a fragment left from an earlier edit. We have removed it. |
| Page14-15: There are two "2.5" in section numbers | The section number has been adjusted to 2.6 |
| Page18: What is the industry sector that this work is focused on? Or is it completely random choice within the region? Is it possible to share the pareto of the rough industry sectors of survey responders? | A section with the industrial sector and professional role of the respondents was added. The section included a table that shows detailed information of the sample’s demographics |
| Page18: What is the definition/criterion of SME and LO in this study? | The definition was based on the one given by the Colombian ministry of commerce. The paragraph has been adjusted accordingly. The following sentences was added : “The Colombian ministry of commerce defines the size of an organization based on the tax value unit (TVU), which is a value equivalent to the Colombian peso used to determine different tax obligations, such as the minimum amounts of withholding at source or penalties. Organizations with a TVU below 1.736.565 are considered SME. All other organizations are ranked as LO.” |
| Page18: How is your survey to SME/LO structured? Can you show the example set of questions? | The Measurement items are shown in Appendix A. |
| Page18: How do the survey responders answer to the questions? Is it based on actual business performance metrics (quantitative)? Or are they based on their impression? This aspect is particularly important to how to interpret the results on page21. | The required information was added:  “Statements related to the operationalization of the various constructs were evaluated based on a five-point Likert-type scale (Strongly Disagree—Strongly Agree) representing the perception of the respondents according to the different questions” |
| Page18: How did you preprocess the survey results to feed them into SPSS/AMOS? Can you show the examples of data processing pipeline or flowchart? | One of the advantages of online survey tools is the ability to transfer the data collected directly to SPSS. We have clarified this thus:  Given the advantages of online surveys (Evans, 2005), we invited potential respondents to participate by email, with a link to access the questionnaire in Microsoft Forms, from which the data can be transferred directly to SPSS. |
| Page18: Is it possible to describe a little more details on SPSS/AMOS parameter settings? | The following section in pages 13 and 14 were improved to address the reviewer’s suggestion.  We used SPSS (IBM Corp) and AMOS software to analyze the data. The analysis stage included confirmation of the conceptualized model shown in Figure 1. The following steps summarize the statistical analysis performed to estimate the model predictiveness as well as its indices.   1. Confirmatory factor analysis (CFA) with the following settings:   • Discrepancy: Asymptotically distribution-free  Covariances supplied as input  • Unbiased  Covariances to be analyzed  • Maximum likelihood   2. Factor loading estimation (assessment of the relationship between the observed and continuous latent variables) 3. Internal consistency (Cronbach’s alpha coefficient shown in table 2) (Cooksey, 2007; Hair et al., 2010). 4. Testing of construct validity (CFA) |
| Page19: Table 3 and it's description seem to fit better after "4. Results and Discussion" | The section containing Tables 2 and 3 was transferred to section 4. Results and discussion |
| Page24: In SME if the outsourcing decision is made by personal interest of the management, where is the assumption coming from? Do you have any data or examples that can be shown? Also does "management's interest" exactly mean? If this is a major factor outsourcing-related responds in the survey seem to be invalid in this study. | Nowhere was this given as a major outsourcing factor. It is mentioned in the conclusion as a possible reason for the results of the study. To clarify our meaning, we have edited the sentence thus:  It is possible that in Colombian SMEs, outsourcing decisions are made based on the personal interests of the management team. |
| Page24-25: The conclusion section is too long, and it is hard to capture the essence of the major discovery. | We have not edited this section as we believe that removing any of the content would detract from the conclusions drawn. |
|  |  |
| Theoretically, the paper was introduced with conceptual framework theorizing the human capital and supply chain drivers in relation with firm’s performance, evidence from specific references from key literatures to support their claims. However reference must be made of recent literatures on the topic which can easily be found and available on google scholars and other search engines. | Following references were added to the document:   - Matthyssens, P. (2019), “Reconceptualizing value innovation for Industry 4.0 and the Industrial Internet of Things”, Journal of Business & Industrial Marketing, Vol. 34 No. 6, pp. 1203-1209. <https://doi.org/10.1108/JBIM-11-2018-0348>” - Falahat, M., Ramayah, T., Soto-Acosta, P., & Lee, Y.-Y. (2020). SMEs internationalization: The role of product innovation, market intelligence, pricing and marketing communication capabilities as drivers of SMEs’ international performance. Technological Forecasting and Social Change, 152, 119908. <https://doi.org/https://doi.org/10.1016/j.techfore.2020.119908>” - Hussain S, Xuetong W, Hussain T. Impact of Skilled and Unskilled Labor on Project Performance Using Structural Equation Modeling Approach. SAGE Open. January 2020. doi:10.1177/2158244020914590 - Manzoor, F., Wei, L., Bányai, T., Nurunnabi, M., & Subhan, Q. A. (2019). An Examination of Sustainable HRM Practices on Job Performance: An Application of Training as a Moderator. Sustainability, 11(8), 2263. - Ferreira, J., Coelho, A., & Moutinho, L. (2020). Dynamic capabilities, creativity and innovation capability and their impact on competitive advantage and firm performance: The moderating role of entrepreneurial orientation. Technovation, 92-93, 102061. <https://doi.org/https://doi.org/10.1016/j.technovation.2018.11.004> - Ho, H, Kuvaas, B. Human resource management systems, employee well-being, and firm performance from the mutual gains and critical perspectives: The well-being paradox. Hum Resour Manage. 2020; 59: 235– 253. <https://doi.org/10.1002/hrm.21990> - Alzabari, S. A. H., Talab, H. R., & Flayyih, H. H. (2019). The effect of internal training and auditing of auditors on supply chain management: An empirical study in listed companies of Iraqi stock exchange for the period 2012-2015. *Int. J Sup. Chain. Mgt Vol*, *8*(5), 1070. - Koot, M., Mes, M. R. K., & Iacob, M. E. (2021). A systematic literature review of supply chain decision making supported by the Internet of Things and Big Data Analytics. Computers & Industrial Engineering, 154, 107076. <https://doi.org/https://doi.org/10.1016/j.cie.2020.107076> - Clauss, T., Abebe, M., Tangpong, C., & Hock, M. (2019). Strategic agility, business model innovation, and firm performance: an empirical investigation. IEEE transactions on engineering management, 68(3), 767-784. - Joiner B. Leadership Agility for Organizational Agility. Journal of Creating Value. 2019;5(2):139-149. doi:10.1177/2394964319868321 - Al Humdan, E., Shi, Y., Behnia, M. and Najmaei, A. (2020), "Supply chain agility: a systematic review of definitions, enablers and performance implications", International Journal of Physical Distribution & Logistics Management, Vol. 50 No. 2, pp. 287-312. <https://doi.org/10.1108/IJPDLM-06-2019-0192> - Abdallah, A.B., Alfar, N.A. and Alhyari, S. (2021), "The effect of supply chain quality management on supply chain performance: the indirect roles of supply chain agility and innovation", International Journal of Physical Distribution & Logistics Management, Vol. 51 No. 7, pp. 785-812. <https://doi.org/10.1108/IJPDLM-01-2020-0011> - Dev, N. K., Shankar, R., & Qaiser, F. H. (2020). Industry 4.0 and circular economy: Operational excellence for sustainable reverse supply chain performance. Resources, Conservation and Recycling, 153, 104583. - Baah, C., Agyeman, D. O., Acquah, I. S. K., Agyabeng-Mensah, Y., Afum, E., Issau, K., ... & Faibil, D. (2021). Effect of information sharing in supply chains: understanding the roles of supply chain visibility, agility, collaboration on supply chain performance. Benchmarking: An International Journal, 29(2), 434-455. - Reschke, Jan, and Sergio Gallego-García. 2021. "A Novel Methodology for Assessing and Modeling Manufacturing Processes" Applied Sciences 11, no. 21: 10117. <https://doi.org/10.3390/app112110117> - Wydyanto, W., & Ilhamalimy, R. R. (2021). Determination of Purchasing Decisions and Customer Satisfaction: Analysis of Service Quality and Product Quality (Marketing Management Literature Review). Dinasti International Journal of Education Management And Social Science, 2(3), 565-575. - Victor DeMiguel, Alberto Martín-Utrera, Francisco J Nogales, Raman Uppal, A Transaction-Cost Perspective on the Multitude of Firm Characteristics, The Review of Financial Studies, Volume 33, Issue 5, May 2020, Pages 2180–2222, <https://doi.org/10.1093/rfs/hhz085> - Tsay, A. A., Gray, J. V., Noh, I. J., & Mahoney, J. T. (2018). A review of production and operations management research on outsourcing in supply chains: Implications for the theory of the firm. Production and Operations Management, 27(7), 1177-1220. |
| The English Language though was fairly good need to be improved. | The manuscript was reviewed by a native English speaker who works for Proofed Inc. |
| Figure 2 was referenced in the results and discussions write up but it cannot be found in the paper. I suggest Figure 3 should rather be labelled as figure 2. | The figure numbers were corrected accordingly. The final structural equation models were labelled as Fig 2 and Fig 3 for SME and LO, respectively |
